# Tomographic, microbiological and histological characterization of secondary apical periodontitis: case series

**DOI:** 10.1590/0103-6440202304590

**Published:** 2023-03-06

**Authors:** Marla Mora-Carabalí, Adolfo Contreras, Patricia Rodríguez, Ingrid Zamora, Martha Rodríguez

**Affiliations:** 1 School of Dentistry, - Faculty of Health, Universidad del Valle, Cali- Colombia.

**Keywords:** secondary apical periodontitis, cone beam computerized tomography, microbial infection, periapical histopathology

## Abstract

This case series included a tomographic, microbiological, and histopathological description of 15 secondary apical periodontitis (SAP) lesions obtained by apical microsurgery performed in 10 patients to better understand the etiology and pathogenesis of SAP. Preoperative tomographic analyses were performed through Cone beam computerized tomography - Periapical index (CBCT-PAI), and apical microsurgeries were then carried out. The removed apices were used for microbial culturing and for molecular identification using PCR for the detection of 5 strict anaerobic bacteria *(P. gingivalis, P. intermedia, P. nigrescens, T. forsythia,* and *T.denticola*) and 3 viruses Herpes simplex viruses (HSV), Cytomegalovirus (CMG) and Epstein-Barr Virus (EBV) by nested PCR. The removed apical lesions were histologically described. Univariate statistical analyses were performed by using STATA MP/16 (StataCorp LLC, College Station, TX, United States). CBCT-PAI analyses revealed PAI 4 and PAI 5 score lesions that involved cortical plate destruction. Eight SAPs were positive by culture, while nine SAP lesions were positive by PCR. *Fusobacterium* species were the most frequently cultured organisms in 7 SAP lesions, followed by *D. pneumosintes* in 3. In contrast, by single PCR, *T. forsythia* and *P. nigrescens* were detected in 5 lesions, *T. denticola* in 4 lesions*,* and *P. gingivalis* in 2 lesions. Twelve periapical lesions were granulomas, and the remaining three SAP lesions were radicular cysts. In conclusion, this case series study revealed that secondary apical lesions presented tomographic involvement of PAI 3 to 5, and that most SAP lesions were apical granulomas containing anaerobic and facultative microorganisms.

## Introduction

Secondary apical periodontitis (SAP) represents a type of endodontic failure in which an apical lesion develops and/or is aggravated after treatment ^1^. SAP develops by persistent and emerging microbial infection of apical tissues with characteristic radiographic findings and is associated with or without symptoms ^2^. SAP lesions also involved an active immune response aimed at limiting apical tissue damage and apical infection. Previous studies identified *E. faecalis. P. alactolyticus, P. propionicum, Parvimonas micra, F. alocis,* and *T. denticola* species in SAP, as well as *Streptococcus, Fusobacterium, Prevotella,* and *Porphyromonas* species ^3^ ^,^ ^4^. This case series of SAP lesions describes their tomographic, microbiological, and histological features to provide some insights into the etiology of and better treatment strategies for SAP.

### Case series

Ten patients aged between 22 and 66 years with posttreatment endodontic diseased teeth who sought treatment at the Endodontics Specialization Program of the Dental School at Universidad del Valle - Cali, Colombia, needing apical surgery were included. This study was approved by the Institutional Human Ethics Review Committee (CIREH) Code #168-2019, and patients were volunteers who signed informed consent forms. The selected subjects had undergone previous endodontic treatment due to i) symptomatic and/or asymptomatic apical periodontitis; ii) chronic apical abscess; and iii) apical surgery indication. A cone-beam computed tomography (CBCT) analysis was performed before and after microsurgery. The lesions and resected apices were processed for microbiological and histopathological analyses as explained below. Data were statistically analyzed with STATA MP/16 (StataCorp LLC, College Station, TX, United States).

### Surgical procedure

At the time of the apical microsurgeries, patients received loco-regional anesthesia with Septocaine® - Articaine HCl 4% and epinephrine 1:100,000 (Septodont Saint-Maur-des-Fossés, France). The required flap design for each case was determined. Complete removal of apical lesion tissue and 3-mm root-end resection was performed in each case. Eather MTA (Angelus S/A, Londrina-PR, Brazil) or Biodentine® (Septodont, Saint-Maur-des-Fossés, France) was used as retrofilling materials, and the suture used in all cases was nylon 5-0 caliber.

### Tomographic analysis:

**Figure 1:**
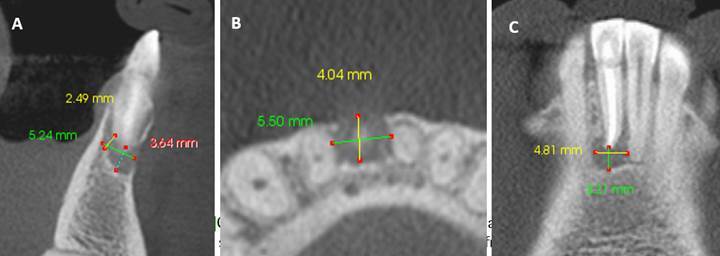
CBCT - PAI 4 +D (destruction), which was the most frequent lesion size. A. Measurements obtained from the sagittal slice; B. Measurements obtained from the axial slice; C. Measurements obtained from the coronal slice.

A CBCT analysis was performed by taking into consideration the classification of the tomographic periapical index (CBCT-PAI), proposed by Estrela et al. ^5^, which describes the severity of periapical lesions in 3 spatial planes and indicates cortical plate compromise. CT scans were analyzed using “In vivo 5 - Anatomage® NVIDIA Corporation software” (Anatomage, Inc. - Santa Clara, CA, United States), which performs 1:1 image compensation, characterizing axial, sagittal, and coronal planes of each tooth and apical lesions, based on the previously mentioned classification, which consists of a score of 1 to 5, 1 being the absence of an apical lesion and 5 being a lesion greater than 8mm. Additionally, it has D for destruction and E for expansion of the cortical plate, as illustrated in Figure 1.

### Microbiological analysis:

Sampling processes: The protocol of Siqueira and Rôças ^6^ and the Bronzato et al. study ^7^ to reduce microbial contamination of the sample were followed and included mouth rinse for 2 minutes with 0.12% chlorhexidine gluconate, followed by skin disinfection with 10% iodopovidone before surgical incision and flap preparation. A high-efficiency vacuum system was used to reduce saliva contamination, and hemostasis was performed with sterile cotton pellets soaked in adrenaline solution to stop the bleeding. The apices of the teeth with SAP lesions were placed into transport media vials containing VGMA III for culture and PCR, and apical tissue was fixed in 10% formalin for histopathological analysis.

### Culture processing and microbial identification of SAP:

An aliquot of VMGA III media containing the SAP lesions was serially plated from undiluted to 10^-5^ dilution in selective trypticase soy agar with bacitracin and vancomycin (Soybean Casein Digest Broth (TSB), Double packed, Comercializadores - Merck S.A., an affiliate of Merck KGaA, Darmstadt, Germany, Bogotá D. C COL) and nonselective Brucella blood agar (BBL Brucella Agar Becton Dickinson Company, Sparks, MD, USA) supplemented with 5% hemolyzed sheep blood with hemin at 5 mg/ml and menadione at 2 mg/ml from the stock solutions, respectively, to a final concentration of 1% in the agar. TSBV was incubated at 37 °C with a 3-5% CO^2^ atmosphere for three days before the analysis, while Brucella blood agar was incubated at 37 °C in anaerobiosis for 14 days using anaerobic jars and Oxoid envelopes. A trained oral microbiologist performed readings, identified colony isolates, and performed further identification of isolates with a rapid ANA test (API 20E, Biomeriux Inc, Marcy lÉtoile, France), catalase, MUG test, CAAM test, and colony PCR test according to Contreras et al. ^8^.

### Molecular microbial detection of SAP:

The molecular analysis of five bacteria and three herpes viruses was performed according to the protocols established in the Operating Procedures of the Oral and Periodontal Microbiology laboratory manual. DNA extractions were performed using the ZYMO Research extraction kit (ZYMO Research Corp., Irvine, CA, United States) according to the manufacturer’s protocol. Tests were performed to verify the quality of DNA extraction from the lesions with two types of genes, namely, the GAPDH gene (to identify human DNA that was positive in the 15 lesions) and bacterial gene rRNA-16S (which was positive in 12/15 lesions). Specific primers described by Ashimoto et al. were used for bacterial species ^9^, while nested PCR according to Contreras et al. ^10^ was used to detect herpes viruses. Boxes 1 and 2 presents the primers and conditions used for the bacteria and viruses.

**Box 1:**
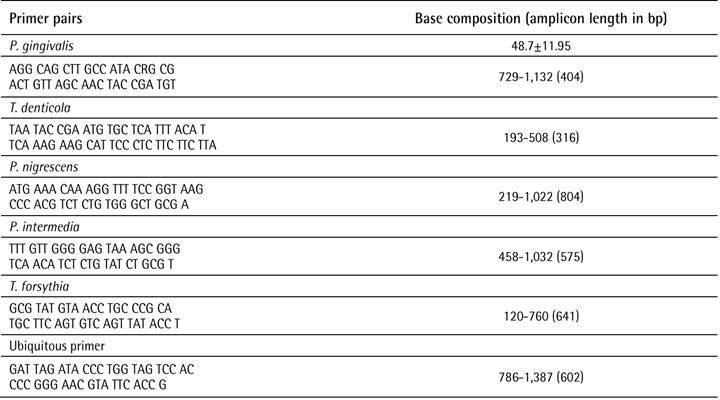
Species-specific and ubiquitous primers used for secondary apical disease.

**Box 2:**
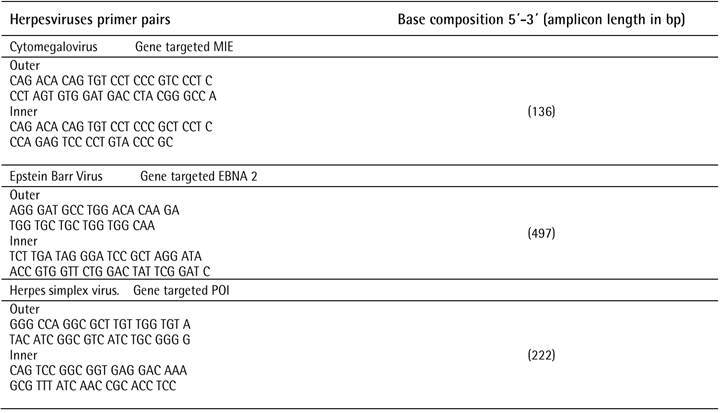
Primers and conditions for viruses.

### Histopathological processing and lesion description of SAP lesions:

**Figure 2:**
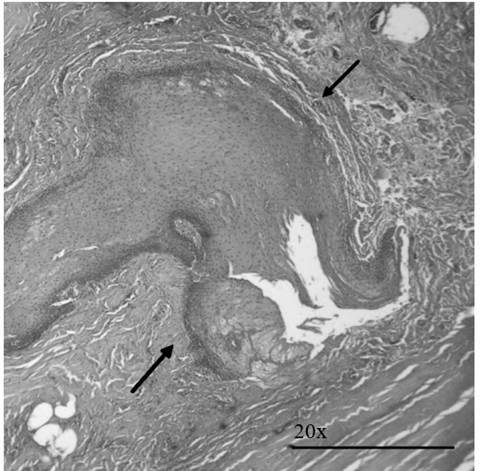
Epithelial inclusion in the connective tissue initiating a radicular cyst. Hematoxylin-eosin staining at 20x. Black arrows indicate the inclusion zone.

Histological processing occurred at the Oral Pathology laboratory and included tissue fixation with 10% formalin for 48 hours, dehydration with alcohol, paraffin inclusion, microtome cutting, and hematoxylin-eosin staining. An experienced oral pathologist, performed histopathological description of SAP lesions, as shown in Figure 2.

### Clinical, tomographic, and sample processing findings

Laboratory data were imported into a Microsoft Excel V16.46 spreadsheet and to STATA MP/16 (StataCorp LLC, College Station, TX, United States), and absolute and relative frequencies of categorical scale variables and summary measures (central tendency, dispersion, and position) of numerical variables were calculated.

Ten subjects, 6 women and 4 men were included, with a mean age of 47.7±11.95 years. The average age of women was higher than that of men (50.33±4.50 and 43.75±18.98 years, respectively), but the differences were not statistically significant (Mann-Whitney test, p=0.5619). Seven subjects belonged to social strata 1 and 2, based on Colombian social strata measure from 1 to 5; 7 also recognized themselves as mestizos, while the remaining 3 were of African descent. Eight patients were ASA I (healthy), and the remaining two were classified as ASA II (moderate systemic disease) (Table 1).

**Table 1:** Sociodemographic description of patients with secondary apical disease.

Variable	Statistical
Age X±DE	48.7±11.95
Female	50.33±4.5
Male	43.75±18.98
Sex n (%)	
Female	6 (60)
Male	4 (40)
Social strata n (%) Greater number indicates higher income	
Level 1	1 (10)
Level 2	5 (50)
Level 3	1 (10)
Level 4	1 (10)
Level 5	2 (20)
Ethnicity n (%)	
Mestizo	8 (80)
African descendent	2 (20)
Educational level n (%)	
Elementary	4 (40)
Technician	1 (10)
College	5 (50)
ASA n (%)	
ASA I	8 (80)
ASA II	2 (20)

Fisher's exact Chi^2^ - 1 side

CBCT PAI scores of 4 and 5 with bone destruction and CBCT PAI scores of 3 and 4 with expansion were the most frequent (Tables 2 and 3). Cases 6, 7 and 13, which had CBCT scores of PAI 5, were histologically classified as periapical granulomas. Cases 6 and 13 also revealed extruded endodontic filling material, a finding confirmed by tomographic analysis and during apical surgery, where gutta-percha debris was evident. Mold hyphae were identified in the sample from case 11 by histopathological analysis. Asymptomatic apical periodontitis was the most frequent clinical diagnosis in 13 cases (Table 2). Twelve apical periodontitis lesions were histologically granulomas, and 3 were radicular cysts. There was no correlation between clinical diagnosis and histopathological diagnosis (exact chi^2^ Fisher=1.000).

The most frequent microorganisms cultured from SAP lesions were *Fusobacterium* species in 7, followed by *Parvimonas micra*, *Campylobacter* species, *Eubacterium* species, and *Dialister pneumosintes* (Table 2). The most frequently identified microorganisms by PCR were *T. forsythia*, *P. nigrescens,* and *T. denticola* (Table 2). Non-herpes viruses were detected in the SAP lesions, and granulomas seem to contain more bacterial species than radicular cysts (Table 3).

The most frequent CBCT-PAI score was 4 +D (destruction) in 4/15 cases (Figure 1). Within these cases, three samples showed a histopathological diagnosis of periapical granuloma, and the fourth sample (from case 14) was a Radicular Cyst; histologically, epithelial inclusion was observed, which initiated the development of the cyst (Figure 2); however, this sample did not show any microbial growth or PCR bacterial presence (Table 3).

**Table 2:** Periapical diagnosis, PAI index and histopathological diagnosis.

		Periapical diagnosis		Histopathological diagnosis		
PAI Score		Asymptomatic apical periodontitis	Chronic apical abscess	Granuloma	Radicular cyst	Total
Expansion (E)	0	0 (0)	0 (0)	0 (0)	0 (0)	0 (0)
	1	1 (14.3)	0 (0)	1 (16.7)	0 (0)	1 (14.3)
	2	1 (14.3)	0 (0)	1 (16.7)	0 (0)	1 (14.3)
	3	2 (28.6)	0 (0)	2 (33.3)	0 (0)	2 (28.6)
	4	3 (42.9)	0 (0)	2 (33.3)	1 (100)	3 (42.9)
	5	0 (0)	0 (0)	0 (0)	0 (0)	0 (0)
Destruction (D)	0	0 (0)	0 (0)	0 (0)	0 (0)	0 (0)
	1	0 (0)	0 (0)	0 (0)	0 (0)	0 (0)
	2	0 (0)	0 (0)	0 (0)	0 (0)	0 (0)
	3	1 (16.7)	0 (0)	0 (0)	1 (50)	1 (12.5)
	4	3 (50)	1 (50)	3 (50)	1 (50)	4 (50)
	5	2 (33.3)	1 (50)	3 (50)	0 (0)	3 (37.5)

Fisher's exact Chi^2^ - 1 side

**Table 3:** Microorganisms identified from secondary apical disease according to histopathology.

Microorganism	Granuloma	Radicular cysts	
	n (%)	n (%)	p
Enteric *Gram* (-) rods	1 (8.3)	1 (33.3)	0.371
*Fusobacterium* spp*.*	4 (33.3)	2 (66.7)	0.341
*Prevotella intermedia/nigrescens*	1 (8.3)	0 (0)	0.800
*Porphyromonas gingivalis*	2 (16.7)	0 (0)	0.629
*Parvimonas micra*	3 (25)	0 (0)	0.484
*Campylobacter* spp*.*	3 (25)	0 (0)	0.484
*Eubacterium* spp.	3 (25)	0 (0)	0.371
*Tannerella forsythia*	0 (0)	0 (0)	-
*Dialister pneumosintes*	3 (25)	0 (0)	0.484
*Streptococci β-hemolytics.*	1 (8.3)	1 (33.3)	0.371
*Streptococci α-hemolytics.*	1 (8.3)	0 (0)	0.800
Yeast	0 (0)	0 (0)	-
*Propionibacterium* spp.	2 (16.7)	0 (0)	0.629
PCR - *Porphyromonas gingivalis*	2 (16.7)	0 (0)	0.629
PCR - *Treponema denticola*	4 (33.3)	0 (0)	0.363
PCR - *Prevotella intermedia*	0 (0)	0 (0)	-
PCR - *Prevotella nigrescens*	5 (41.7)	0(0)	0.242
PCR - *Tannerella forsythia*	4 (33.3)	1 (33.3)	0.242
PCR - HSV1	0 (0)	0 (0)	-
PCR - CMV	0 (0)	0 (0)	-
PCR - EBV	0 (0)	0 (0)	-

Fisher's exact Chi^2^ - 1 side

## Discussion

CBCT represents an important diagnostic technology for identifying the complexity and extension of apical lesions, allowing clinicians to define better treatment guidelines ^11^. Estrela’s CBCT-PAI classification method ^5^ can determine lesion severity and prognosis and lead to treatment decisions in apical periodontitis ^12^ ^,^ ^13^. In the present case series study, apical lesions were evaluated by CBCT-PAI prior to endodontic surgery to determine cortical perforation or cortex expansion. Interestingly, the SAP lesions with higher CBCT-PAI also harbored polymicrobial infections and were granulomas, as seen in Tables 2 and 3. Most SAP lesions were described in our study as CBCT-PAI 4 +D (destruction) and PAI 5 +D, followed by PAI 3 and PAI 4 with expansion (+E). Some studies have attempted to relate histopathological diagnoses of apical lesions with CBCT density using Hounsfield Units (HU). However, as Pauwels et al. mentioned in their study, HU is not applicable to CBCT ^14^.

The most frequent periapical diagnosis in this case series of SAP lesions was asymptomatic apical periodontitis. Interestingly, studies related to post-endodontic treatment and apical periodontitis did not usually detail the diagnosis ^15^; therefore, determining the size, origin and histopathology of SAP lesions is crucial for treatment planning and prognosis prediction ^3^.

SAP is caused by microorganisms organized in a biofilm inside and outside of the root canal system ^16^. Microbial identification is essential to understand inflammatory and immune reactions derived from SAP ^1^. The most frequent species identified in the present study were *Fusobacterium* species, *Parvimonas micra*, *Campylobacter* species, *Eubacterium* species, and *Dialister pneumosintes* by culture and *T. forsythia* and *P. nigrescens* followed by *Treponema denticola* by PCR (Table 4), findings that are consistent with other SAP studies ^7^ ^,^ ^17^ ^-^ ^19^.

**Table 4:** Microorganisms identified in secondary apical endodontic lesions.

Microorganisms/Sample	1	2	3	4	5	6	7	8	9	10	11	12	13	14	15
Enteric *Gram* (-) rods					x				x						x
*Fusobacterium* spp					x	x		x	x	x	x				x
*Prevotella intermedia/nigrescens*								x							
*Porphyromonas gingivalis*								x		x					
*Parvimonas micra*								x	x						x
*Campylobacter* spp						x		x		x					
*Eubacterium* spp						x			x	x					
*Dialister pneumosintes*						x			x	x					
*Streptococci β-hemolytics.*											x	x			
*Streptococci α-hemolytics.*								x							
*Propionibacterium* spp*.*							x		x						
*Eikenella corrodens*															
*Tannerella forsythia*															
*Yeast*															
PCR *T. forsythia*					x	x	x				x				x
PCR *P. nigrescens*		x	x	x		x		x							
PCR T*. denticola*	x									x	x		x		
PCR *P. gingivalis*	x														x
PCR *P. intermedia*															

The genus *Fusobacterium* is an intermediate colonizer of the oral biofilm that allows the adhesion of late colonizers (16). Some species of *Parvimonas micra*, *Campylobacter* species, *Eubacterium* species, *Dialister pneumosintes*, and *α* and *β* hemolyti*c Streptococci* were also present in the study with low frequencies, which is consistent with findings in the literature ^6^ ^,^ ^7^.

There are previous reports of mainly gram-positive facultative anaerobic microorganisms being predominant in SAP lesions ^4^ ^,^ ^6^, although it was also considered that gram-negative facultative anaerobes are also abundant ^20^. Streptococcus species comprise 9% to 99% of the total bacteria in endodontically treated teeth ^21^. Regarding abundance, streptococci can be considered to play an important role in endodontic failures ^19^. α and β hemolytic *Streptococci* in the current study confirmed that finding with their low prevalence, as depicted in Tables 3 and 4.

Molecular detection results were consistent with those of other studies ^7^ ^,^ ^19^. *T. forsythia*, *P. nigrescens*, and *T. denticola* detection rates were similar to those in Siqueira’s study, in which specimens were collected from apical surgeries in endodontically treated teeth and cryogenically powdered. Then, DNA was extracted from the powder, and the microbiome was characterized by 16S rRNA gene paired-end sequencing. The most abundant phylotypes detected in Siqueira’s study were *Proteobacteria*, *Firmicutes, Fusobacteria,* and *Actinobacteria,* and the most common genera were *Fusobacterium*, *Pseudomonas*, *Treponema, Tannerella,* and *Porphyromonas* ^20^. Although *Fusobacterium* species were not evaluated in our study by molecular testing, they were identified by culture, as depicted in Table 4. The absence of *P. intermedia* in our case series study may be related to some variation among diverse populations. Siqueira et al. ^22^ determined the microbial composition in 28 teeth with apical periodontitis, and *P. nigrescens* was only detected in 2 lesions. In contrast, we detected *P. nigrescens* in 5 lesions (33.3%), as shown in Tables 3 and 4. Table 4 indicates the microorganisms identified in each SAP lesion and reveals that molecular detection is more sensitive than culture.

Regarding viruses, studies on periapical disease are still scarce. However, in those that have been analyzed, the prevalence of herpes simplex virus, cytomegalovirus, and Epstein-Barr virus was relatively high ^4^ ^,^ ^23^. However, contrary to these studies, the presence of herpesviruses was negative in the 15 SAP patients even though the very sensitive nested PCR technique was used ^9^.

Histological studies in endodontics have been performed to provide an important basis for understanding the nature of the disease according to clinical signs and symptoms, as well as the treatment approaches for inflammatory processes ^24^. In the present study, periapical granulomas were the most frequent diagnosis (80% of SAP cases), consistent with the findings in the literature noted by Ricucci et al. and Gbadebo et al. ^25^ ^,^ ^26^. Radicular cysts were reported in the other 3 SAP lesions and perhaps represent another type of host response with the aim of isolating a lesion in an active endodontic infection ^25^. Interestingly, those types of lesions harbored fewer microorganisms, as depicted in Table 3.

In the study by Gbadebo et al. ^26^, 19 cases were analyzed. Clinically, 13 cases (68.4%) were diagnosed as radicular cysts. However, histopathology revealed that 16 (84.4%) were radicular cysts. On 13 of the clinically and radiographically classified cysts, a sclerotic border was found, which refers to epithelialization as mentioned by Nair in 2004 ^1^. This analysis revealed how complex it is to clinically identify a lesion.

The presence of foreign bodies on histopathological analyses is associated with the development of apical lesions and is known as a nonmicrobial cause of SAP ^27^ ^,^ ^28^. The histopathological findings here indicated the presence of a mixed inflammatory cellular infiltrate composed of acute and chronic inflammatory cells associated with foreign bodies. However, the impact of endodontic pathogens on SAP lesion development cannot be ruled out.

One SAP lesion had fungal hyphae in the extra radicular zone, as identified by histopathology and described by Waltimo et al. ^29^, which highlighted the limitations of intracanal endodontic therapy and the importance of endodontic surgery to treat apical granulomas and radicular cysts. Apical lesions can be considered scar tissue, as reported by Nair ^30^ and Çalışkan ^31^. It is important to note that some SAPs can be related to *E. faecalis* infection ^15^ ^,^ ^16^. However, we did not perform specific detection for this important endodontic pathogen.

In conclusion, this case series study revealed that secondary apical lesions presented tomographic involvement of PAI 3 to 5, and that most SAP lesions were apical granulomas containing anaerobic and facultative microorganisms. These findings aim to facilitate better recognition of the pathogenesis of secondary apical disease to develop better prevention and treatment plans.
